# Professional Ethics and Preserving Human Dignity in Clinical Laboratories

**DOI:** 10.30699/ijp.2025.2064314.3483

**Published:** 2025-11-08

**Authors:** Alireza Abdollahi, Mohammad Reza Jalali Nadoushan

**Affiliations:** 1 *Department of Pathology, School of Medicine, Imam Khomeini Hospital Complex, Tehran, Iran*; 2 *Associate Editor, Iranian Journal of Pathology, Tehran, Iran*; 3 *Dept. of Pathology, Shahed University, Tehran, Iran*; 4 *Editor-in-Chief, Iranian Journal of Pathology, Tehran, Iran*

In an era where precision medicine and technological advancements define modern healthcare, the importance of maintaining ethical standards and human dignity within clinical settings remains paramount. Clinical laboratories, as fundamental components of the diagnostic process, often operate behind the scenes, yet their influence on patient care is direct and significant. Ethical conduct in these settings can shape patients' trust, influence compliance, and ultimately impact clinical outcomes (1).

Patients entering laboratories are often anxious, vulnerable, and in search of answers to potentially life-altering questions. Even brief interactions with laboratory personnel can leave lasting impressions. Professional ethics in this context extend beyond technical accuracy to include respect, confidentiality, empathy, and equitable treatment.

Human dignity, as a cornerstone of medical ethics, can be challenged in fast-paced laboratory environments. The risk of depersonalized care is particularly high in laboratories focused solely on efficiency and throughput. However, patients are not merely sample sources—they are individuals with unique needs, fears, and rights (2). Upholding their dignity reinforces a humanistic approach to medicine and aligns with international ethical standards (3).

Professional ethics in laboratories encompass the following core dimensions:

- Respect for autonomy and privacy: Patients should be informed about procedures, and consent must be obtained for invasive sampling. Maintaining patient privacy is important from two perspectives: physical and psychological. Maintaining physical distance from the patient during sampling is very important, especially considering the cultural and religious beliefs of the individuals. It is also important not to ask questions and issues unrelated to the patient's tests or political/ideological conversations, and to maintain their psychological and mental security.

- Confidentiality and data security: Results must be securely stored and shared only with authorized parties (4). Avoid talking about the patient's condition in public, and do not give them unnecessary medical information that is not within the scope of the laboratory's work.

- Equity and non-discrimination: All individuals must be treated equally, regardless of gender, age, ethnicity, or social status. Discrimination in service provision or its quality violates human dignity.

- Empathetic communication: Laboratory staff should be trained to communicate with compassion and cultural sensitivity.

Scientific Precision and Responsibility: Accurate and precise testing with standard recording of results is the right of the patient. Errors in reporting results can have serious consequences and are considered a breach of patient trust.

## Effects of Upholding Ethics and Dignity

The benefits of maintaining professional ethics and human dignity are both tangible and intangible. On the one hand, patients experience reduced anxiety, higher satisfaction, and increased trust in the healthcare system. On the other hand, laboratories benefit from improved reputation, fewer complaints or legal disputes, and greater staff morale and professional pride (1)(5). Ethical practice contributes to institutional integrity and fosters a culture of respect and accountability. Smiling gently and reassuring the patient with body language has a great impact on creating peace in the patient and trust in the laboratory.

## Strategies for Enhancing Ethical Standards

1. Continuous education and training: Ethics, empathy, and communication skills should be core elements of ongoing professional development for laboratory staff. Role-playing, workshops, and reflective practices can help build awareness and skills (2).
2. Development of a Patient’s Bill of Rights: Posting clear and accessible information about patient rights in the laboratory environment enhances transparency and reinforces institutional commitment to dignity.
3. Structured patient feedback systems: Anonymous feedback forms, online surveys, and real-time satisfaction metrics can highlight areas of concern and promote responsive improvements.
4. Designing human-centered physical spaces: Facilities should prioritize privacy, especially during sample collection. Simple adjustments like privacy screens, comfortable waiting areas, and noise control can dramatically improve the patient experience (3).
5. Leadership modeling and ethical governance: Ethical conduct should be promoted from the top, with supervisors exemplifying best practices and recognizing staff who uphold these values.
6. Special accommodations for vulnerable populations: Children, the elderly, individuals with disabilities, and non-native language speakers require tailored approaches, including extra time, clearer instructions, and visual aids (4).
7. Enforcing confidentiality and digital security protocols: Modern laboratories must ensure that electronic health records are secure, with staff trained to avoid inadvertent data breaches.
8. Organizational commitment: Laboratory employees must be loyal to their organization and do not have the right to speak ill of the system and other colleagues in front of patients. Rather, they must work with the authorities to eliminate the organization's errors and shortcomings.
9. Maintaining the human dignity of employees: It is very important that employees feel valued in the workplace and that their human dignity is respected by officials and managers. How to deal with personnel errors in a way that both preserves their human dignity and provides feedback appropriate to the type of error to prevent its recurrence is one of the abilities of a successful and knowledgeable manager.

Finally, it can be concluded that Laboratories must go beyond delivering accurate results—they must embody the principles of ethical care and human dignity. By prioritizing respect, empathy, and patient-centeredness, they enhance both the technical and human quality of healthcare. Ethical practices build a foundation of trust and professionalism, benefiting patients, staff, and the health system as a whole. Organizations are more successful when they tap into their customers' emotions by communicating effectively and empathetically.

Ultimately, honoring human dignity in laboratories is not merely a moral ideal; it is a practical imperative that yields emotional, professional, and institutional rewards.
